# Supplementary material for: Lapsyn controls branch extension and positioning of astrocyte-like glia in the Drosophila optic lobe
Source: Nat Commun. 2017 Aug 22;8:317. doi: 10.1038/s41467-017-00384-z (PMC5567088; doi:10.1038/s41467-017-00384-z)
Supplement: Supplementary file 1 — Supplementary Information [file 41467_2017_384_MOESM1_ESM.pdf]

File Name: Supplementary Information
Description: Supplementary Figures, Supplementary Tables and Supplementary References.

File Name: Supplementary Data 1
Description: Supplementary Data 1 contains the source data used for quantifications shown in Figs. 2, 4-8, and Supplementary Fig. 7.

File Name: Peer Review File
Description:

Supplementary Figure 1

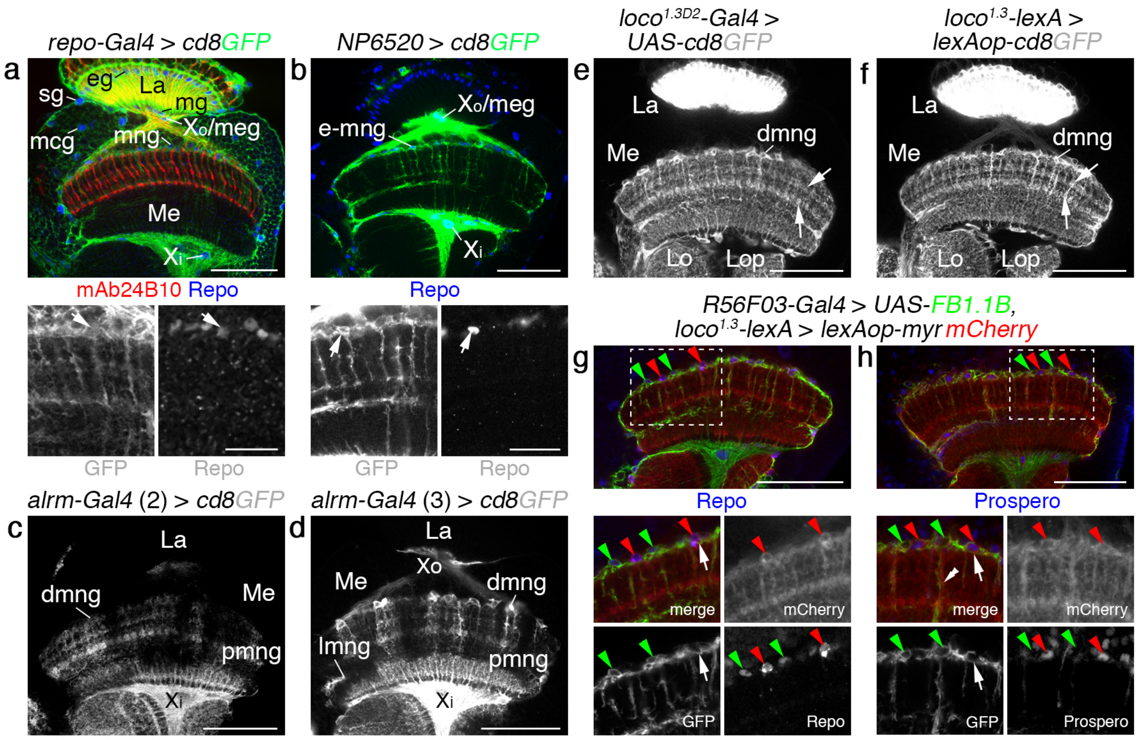

Supplementary Figure 1 | Additional characterization of glial drivers in the adult visual system. (**a**) *repo-Gal4* drives expression of *UAS-cd8GFP* (green) in all glial cell types in the lamina (La) and medulla (Me), including epithelial glia (eg), marginal glia (mg), medulla glia in the outer chiasm (Xo, meg), surface glia (sg), medulla cortex glia (mcg), medulla neuropil glia (mng) and glia in the inner chiasm (Xi). *repo-Gal4* activity appears higher in ensheathing (e-mng) than astrocyte-like mng. Glial nuclei were labeled with Repo (blue). (**b**) *NP6250-Gal4* drives expression of *UAS-cd8GFP* in e-mng, as well as in Xo and Xi chiasm glia. The strength of this driver varies between samples. (**c**,**d**) *alrm-Gal4* insertions on the 2nd (**c**) and 3rd (**d**) chromosomes drive mosaic expression of *UAS-cd8GFP* (white) in distal, proximal and lateral astrocyte-like medulla neuropil glia (dmng, pmng and lmng) in the adult medulla. *alrm*-Gal4 is not active in epithelial and marginal glia in the lamina. *alrm*-Gal4 labels outer and inner chiasm glia (Xo and Xi). (**e**) *loco*$^{1.3D2}$*-Gal4* drives expression of *UAS-cd8GFP* (green) in mng, whose processes show distinct layered branching patterns (arrows) in the adult medulla neuropil. (**f**) The newly generated *loco*$^{1.3}$*-lexA* transgene drives expression of *lexAop-cd8GFP* in a similar pattern as the *Gal4/UAS* binary system in the adult visual system. (**g**,**h**) Combining *loco*$^{1.3}$*-lexA* driving *lexAop-cd8mCherry* (red) in astrocyte-like mng (**g**, red arrowhead) with *R56F03-Gal4* driving *UAS-FB1.1B*^{260b} (green) in e-mng in the same sample confirms their expression in distinct glial cell subtypes (**g**, red and green arrowheads). Glial nuclei were labeled with Repo (**g**, blue). Astrocyte-like mng (red arrowheads), but not e-mng (green arrowheads) express Prospero (**h**, blue). Branches of e-mng and astrocyte-like mng extending into the neuropil are found in close physical contact (**h**, double arrowhead). At the medulla neuropil border, e-mng processes encircle the cell bodies of astrocyte-like mng (**g**,**h**, arrow). All panels show single optical sections. For genotypes and sample numbers, see Supplementary Table 1. Scale bars, 50 µm.

Supplementary Figure 2

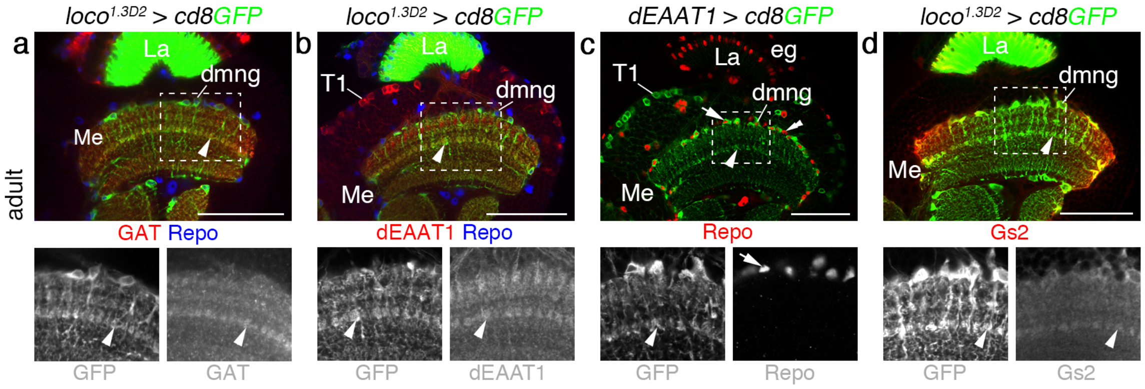

Supplementary Figure 2 | Molecular profile of adult astrocyte-like medulla neuropil glia (mng). (**a**,**b**,**d**) *loco*$^{1.3D2}$*-Gal4* drives expression of *UAS-cd8GFP* (green) in astrocyte-like mng including distal mng (dmng). Glial nuclei were labeled with Repo (blue, **a**,**b**; red, **c**). Immunolabeling with antibodies against the GABA transporter, GAT (red) (**a**), the excitatory amino acid transporter 1, dEAAT1 (red) (**b**) and the glutamate synthetase 2, Gs2 (red) (**d**) reveal expression in branches of astrocyte-like mng (arrowhead). (**c**) Similarly, *dEAAT1-Gal4* drives expression of *UAS-cd8GFP* (green) in cell bodies (arrow) and processes (arrows) of astrocyte-like mng, but not in ensheathing glia (double arrowhead). dEAAT1 is also expressed in T1 neurons (**b**,**c**). All panels show single optical sections. For genotypes and sample numbers, see Supplementary Table 1. Scale bars, 50 μm.

Supplementary Figure 3

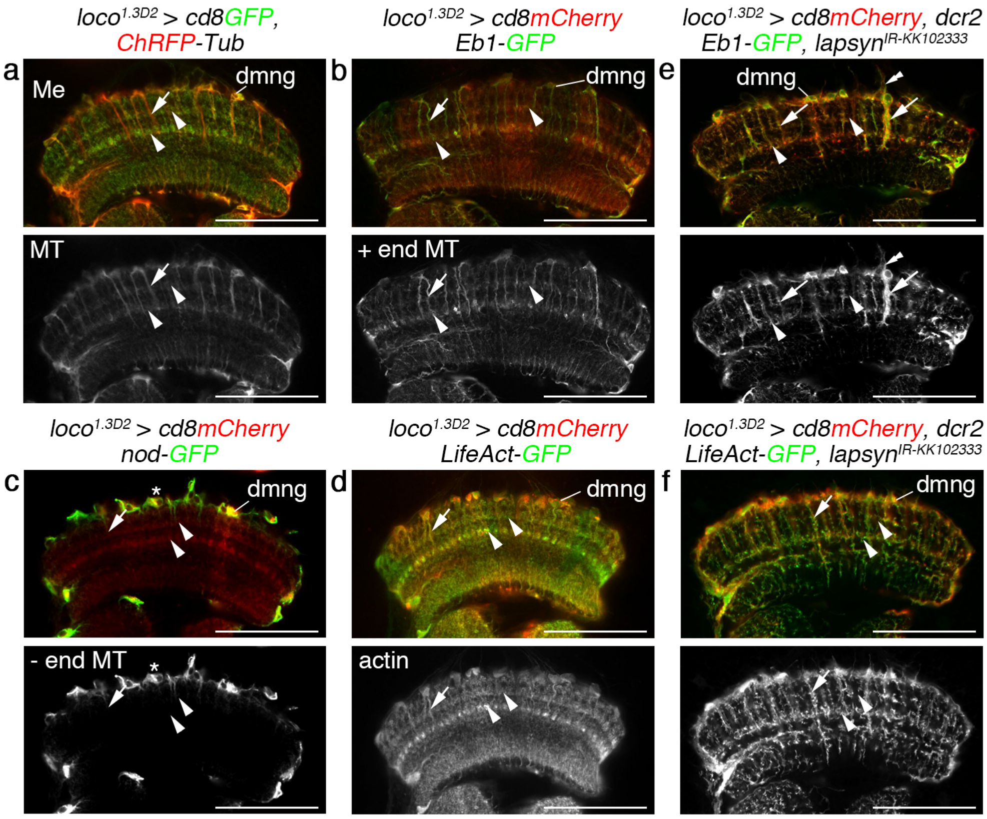

Supplementary Figure 3 | Effects of *lapsyn* knockdown on the actin and microtubule cytoskeleton. (**a-f**) Expression of RFP (red) or GFP (green)-tagged versions of cytoskeletal proteins in astrocyte-like medulla neuropil glia (mng) labeled with *loco*$^{1.3D2}$*-Gal4 UAS-cd8GFP* (green) or *loco*$^{1.3D2}$*-Gal4 UAS-cd8mCherry* (red), respectively. Arrows indicate expression in primary branches, arrowheads indicate expression in secondary branches in medulla neuropil layers M3 and M5. (**a**) *ChRFP-Tub* reveals an enrichment of microtubules (MT) in primary branches. (**b**,**c**) The microtubule plus-end marker *Eb1-GFP* and the minus-end marker *nod-GFP* show a polarized distribution: *Eb1-GFP* is located mainly in primary main branches (**b**); *nod-GFP* is enriched in cell bodies (asterisk) and absent in branches (**c**). (**d**) *LifeAct-GFP* reveals an abundance of actin filaments in primary branches and secondary branches. (**e**,**f**) Knockdown of *lapsyn* results in impaired mng branching and in the disorganization of the actin and microtubule cytoskeleton. (**e**) Microtubules are detected in primary and truncated secondary branches, as well as in short processes extending into the cortex (double arrowheads). (**f**) Actin filaments are mostly found in primary branches of astrocyte-like mng and appear punctated, consistent with a substantial loss of mng secondary branches. All panels show single optical sections. For genotypes and sample numbers, see Supplementary Table 1. Scale bars, 50 µm.

Supplementary Figure 4

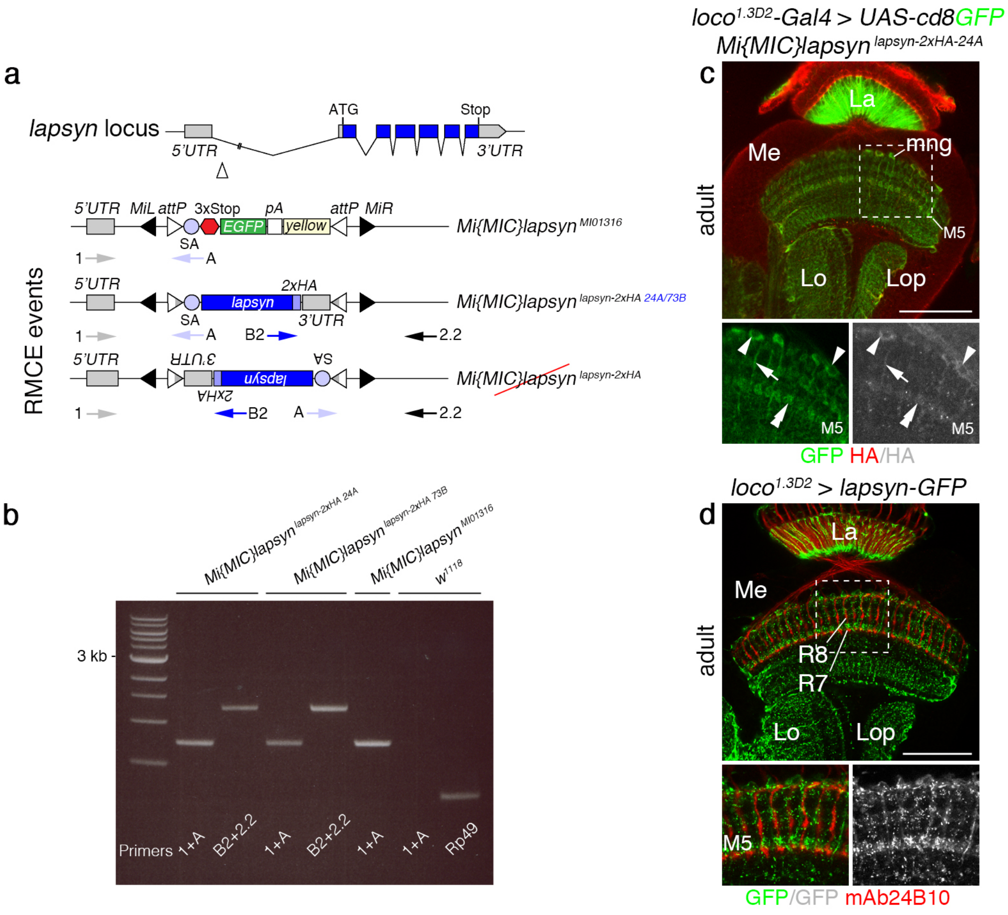

Supplementary Figure 4 | Further expression analysis of *lapsyn*. (**a**) RMCE was used to integrate *lapsyn* cDNA via the *attP* sites of *Mi{MIC}lapsyn*^{MI01316} into the *lapsyn* genomic locus to generate *Mi{MIC}lapsyn*$^{lapsyn\text{-}2xHA}$. RMCE events within *Mi{MIC}lapsyn*^{MI01316} can result in the integration of a replacement cassette in the correct 5'-3' or incorrect 3'-5' orientation. *MiL*, *MiR* Minos inverted repeats; SA, splice acceptor site; Stop, 3 stop codons; *EGFP*, cDNA encoding enhanced green fluorescent protein; pA, polyadenylation signal; *yellow*, *yellow*$^{+}$ marker. PCR reactions used the RMCE primer pairs 1 and A, as well as B2 and 2.2 (arrows). (**b**) Validation of RMCE-mediated integration of *Mi{MIC}lapsyn*^{lapsyn2xHA} insertions. PCR amplification of genomic DNA from 30 flies were performed for *Mi{MIC}lapsyn*$^{lapsyn2xHA\text{-}24A}$*/CyO, Mi{MIC}lapsyn*$^{lapsyn2xHA\text{-}73BA}$*/CyO, Mi{MIC}lapsyn*^{MI01316}/CyO *and w*^{1118} strains. Bands matching the expected sizes of 730 bp and 1216 bp demonstrated that the cassette integrated correctly. PCR amplification of a 240 bp *Rp49* fragment in the *w*^{1118} strain served as a positive control. (**c**) *Mi{MIC}lapsyn*$^{lapsyn\text{-}2xHA\text{-}24A}$ reports Lapsyn protein localization (red) in astrocyte-like medulla neuropil (mng) cell bodies (arrowheads), primary (arrows) and secondary (double arrowheads) processes in layer M5. (**d**) *UAS-lapsyn-GFP* over-expression by *loco*$^{1.3D2}$*-Gal4* shows punctate Lapsyn protein in mng processes. R-cell axons were labeled with mAb24B10 (red). La, lamina; Lo, lobula; Lop, lobula plate; Me, medulla. All image panels show single optical sections. For genotypes and sample numbers, see Supplementary Table 1. Scale bars, 50 μm.

Supplementary Figure 5

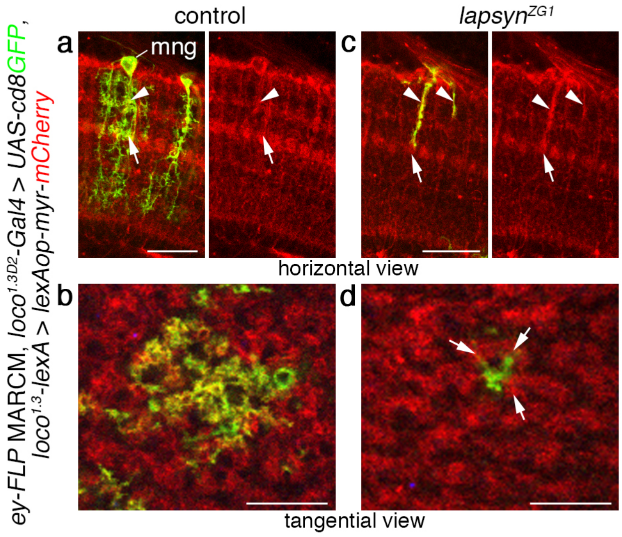

Supplementary Figure 5 | Compensatory neuropil coverage by astrocyte-like medulla neuropil glia (mng) adjacent to *lapsyn* deficient mng. (**a**–**d**) All astrocyte-like mng were labeled with *loco*$^{1.3}$-*LexA LexAop-myr mCherry* (red). Control (**a**,**b**) and *lapsyn*^{ZG1} (**c**,**d**) *ey-FLP* MARCM clones also expressed *loco*$^{1.3D2}$-*Gal4 UAS-cd8GFP* (green, arrowheads). Compared to controls (**a**, arrows), reduced neuropil coverage by *lapsyn*^{ZG1} astrocyte-like mng branches is covered by processes of adjacent heterozygous mng (**c**,**d**, arrows). Optic lobes are shown in horizontal (**a**,**c**) and tangential (**b**,**d**) views. La, lamina; Lo, lobula; Lop, lobula plate; Me, medulla. All panels show single optical sections. For genotypes and sample numbers, see Supplementary Table 1. Scale bars, 50 μm (**a**,**b**), 10 μm (**c**,**d**).

Supplementary Figure 6

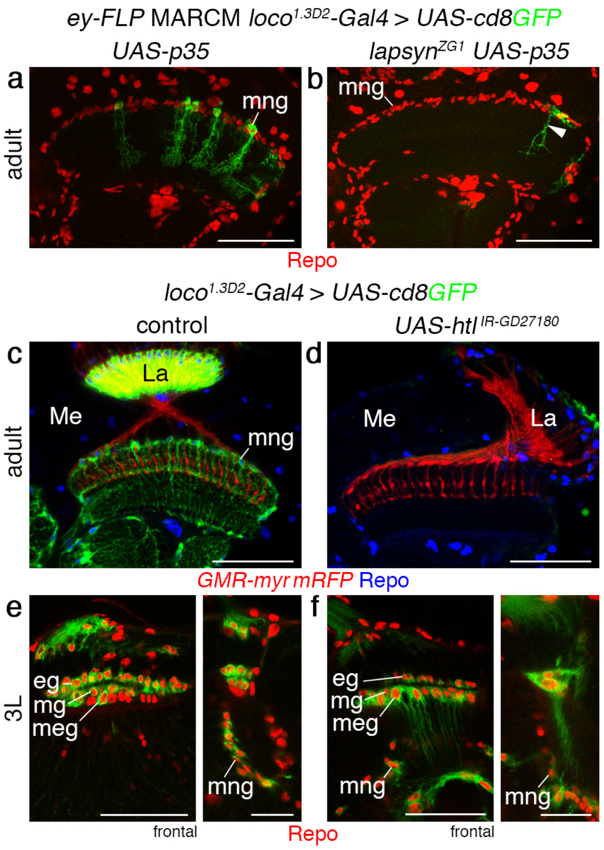

Supplementary Figure 6 | Further assessment of medulla neuropil glial apoptosis phenotypes. (**a**,**b**) Control and *lapsyn*^{ZG1} mng clones over-expressing *UAS-p35* were generated using *ey-FLP* in conjunction with *loco*$^{1.3D2}$*-Gal4 UAS-cd8GFP* (green). Glial nuclei were labeled with Repo (red). Over-expression of *UAS-p35* did not cause any defects (**a**) and did not rescue mng cell numbers and branching defects (arrowhead) caused by the loss of *lapsyn*^{ZG1} (**b**). (**c**–**f**) *loco*$^{1.3D2}$*-Gal4* drives expression of *UAS-cd8GFP* (green) in astrocyte-like medulla neuropil glia (mng). R-cell axons were labeled with *GMR-RFP* (**c**,**d**, red) and glial nuclei with Repo (**c**,**d**, blue; **e**,**f**, red). Persistent expression of *UAS-htl*$^{IR\text{-}GD27180}$ with *loco*$^{1.3D2}$*-Gal4* at 29°C caused severe loss of glia in the lamina (La) and astrocyte-like mng in the medulla (Me) of adult flies (**d**) compared to controls (**c**). (**e**,**f**) In third instar larval (3L) optic lobes, epithelial (eg) and marginal glia (mg) in the lamina and medulla glia (meg) and mng form, but compared to controls (**e**) cell bodies are small and appear to undergo apoptosis upon early *htl* knockdown (**f**). (**e**,**f**, right hand panels) Larval optic lobes are shown in frontal and horizontal orientations. Panels **a**,**b** show projections, panels **c**–**f** single optical sections. For genotypes and sample numbers, see Supplementary Table 1. Scale bars, 50 μm (**a**–**d** and **e**,**f** left hand panels), 25 μm (**e**,**f** right hand panels).

Supplementary Figure 7

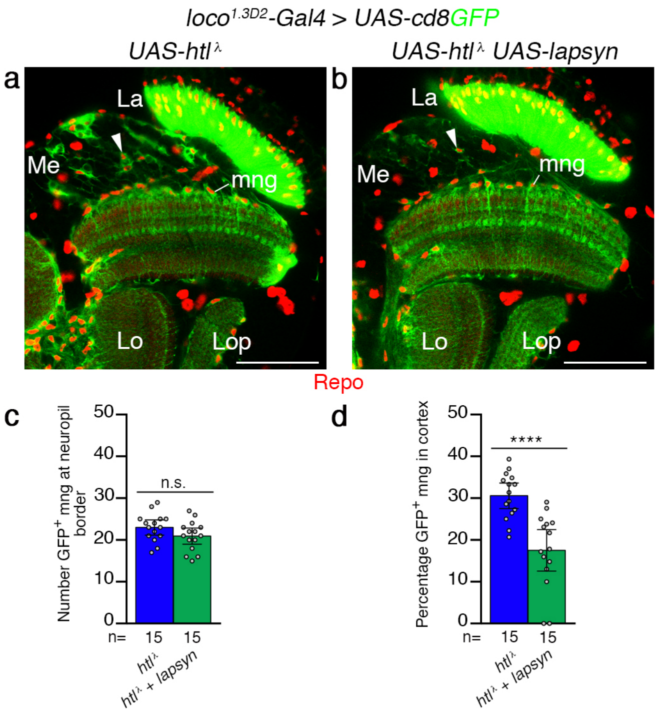

Supplementary Figure 7 | Effects of co-expression of *lapsyn* and constitutively active *heartless.* (**a**,**b**) *loco$^{1.3D2}$-Gal4* is used to co-express *UAS-cd8GFP* (green) with *UAS-htl$^{\lambda}$* (**a**) or *UAS-htl$^{\lambda}$* and *UAS-lapsyn* (**b**) in astrocyte-like medulla neuropil glia (mng) in the adult medulla (Me). Increased expression of *lapsyn* reduced the number of ectopically positioned mng (arrowheads) in the medulla cortex. La, lamina; Lo, Lobula; Lop, Lobula plate. (**c**) In quantifications of GFP-positive mng per optical section, the total number of dmng, lmng and pmng was not significantly different in flies over-expressing *UAS-htl$^{\lambda}$* or *UAS-htl$^{\lambda}$* and *UAS-lapsyn*. (**d**) In quantifications of the percentage of GFP-positive mng detected in the medulla cortex, over-expression of *lapsyn* reduced the percentage of ectopic mng in the adult medulla cortex relative to the total number mng (in cortex, dmng, lmng and pmng) by 43%. Scatter plots with bars show data points and means ± 95% confidence intervals. (n=15 optical sections from 3 optic lobes per genotype). Unpaired, two-tailed Student's *t* test not assuming equal variance: (**c**) *P*=0.1109, (**d**) P=7.35x10^{-5}. n.s., not significant, ****P<0.0001. Panels **a**,**b** show single optical sections. For genotypes, sample numbers and additional statistical values, see Supplementary Tables 1 and 2. Scale bars, 50 µm.

Supplementary Figure 8

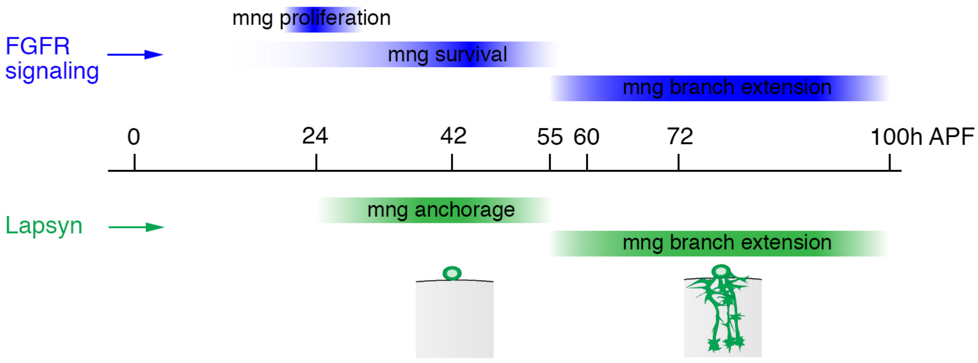

Supplementary Figure 8 | Model illustrating the roles of *lapsyn* relative to the FGF signaling pathway in controlling astrocyte-like medulla neuropil glia (mng) development. During early pupal development, FGF signaling promotes the proliferation and survival of astrocyte-like mng at the medulla neuropil border. *lapsyn* promotes mng anchoring at the border to reduce their migration into the cortex, consequently ensuring their survival by exposure to sufficient gliotrophic FGF signaling. From mid-pupal development, *lapsyn* and the FGF signaling pathway act in parallel to control robust glial branch morphogenesis.

Supplementary Figure 9

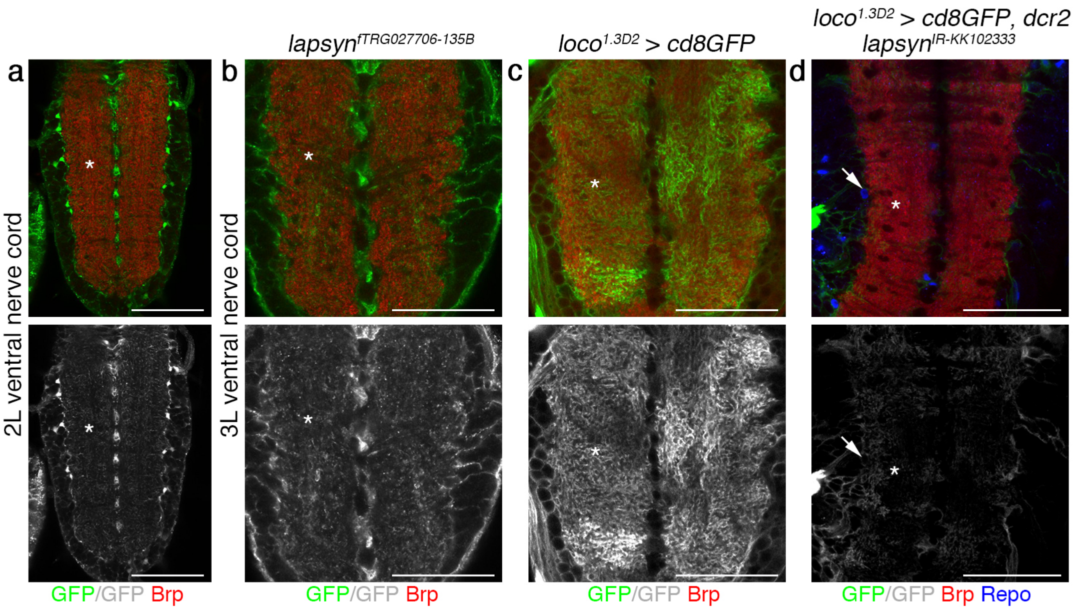

Supplementary Figure 9 | Effects of *lapsyn* knockdown in the larval VNC. Brp was used to label the synaptic neuropil (red, **a**–**d**). (**a**,**b**) The fosmid *lapsyn*^{fTRG027706} reported Lapsyn protein expression (green) in the processes of astrocyte-like glia (asterisks) and other glial subtypes in the second instar (2L) and wandering third instar (3L) larval VNC. (**c**,**d**) Astrocyte-like glial processes, labeled with *loco*$^{1.3D2}$*-Gal4 UAS-cd8GFP*, abundantly infiltrated the larval VNC neuropil (green, asterisks). Compared to controls (**c**), the knockdown of *lapsyn* (**d**) strongly reduced branch extension (asterisks) by astrocyte-like glia located at the neuropil border (arrow). Glial nuclei were labeled with Repo (blue, **d**). Panels show single optical sections. For genotypes and sample numbers, see Supplementary Table 1. Scale bars, 50 μm.

Supplementary Table 1 | Full genotypes and numbers of samples shown in main and supplementary figure panels

Figure	Panel	Genotype	n=
**Fig. 1**	**b**	R56F03-Gal4/+; UAS-cd8GFP/+	9
	**c**	GMR-myrRFP/+ or Y; loco$^{1.3D2}$-Gal4/+; UAS-cd8GFP/+	25
	**d–j**	loco$^{1.3D2}$-Gal4/UAS-FB1.1^{260b}; hs-mFLP5/+	60[a]
	**k–n**	loco$^{1.3D2}$-Gal4/UAS-FB1.1^{260b}; hs-mFLP5/+	8
**Fig. 2**	**b**	FRT19A tubP-Gal80 hs-FLP^{1}/FRT19A; UAS-lacZ^{nls}, UAS-cd8GFP/+; tub-Gal4/+	18
	**c**	FRT19A tubP-Gal80 hs-FLP^{1}/FRT19A; UAS-lacZ^{nls}, UAS-cd8GFP/+; tub-Gal4/+	6
	**d**	FRT19A tubP-Gal80 hs-FLP^{1}/FRT19A; UAS-lacZ^{nls}, UAS-cd8GFP/+; tub-Gal4/+	11
	**e**	FRT19A tubP-Gal80 hs-FLP^{1}/FRT19A; UAS-lacZ^{nls}, UAS-cd8GFP/+; tub-Gal4/+	35
	**f**	loco$^{1.3D2}$-Gal4; UAS-cd8GFP	9
	**i**	GMR-myr mRFP; loco$^{1.3D2}$-Gal4; UAS-cd8GFP, UAS-dcr2	12
	**j**	GMR-myr mRFP; loco$^{1.3D2}$-Gal4; UAS-cd8GFP, UAS-dcr2	16
	**k**	GMR-myr mRFP; loco$^{1.3D2}$-Gal4; UAS-cd8GFP, UAS-dcr2	10
	**l**	GMR-myr mRFP; loco$^{1.3D2}$-Gal4; UAS-cd8GFP, UAS-dcr2	12
	**m**	GMR-myr mRFP; loco$^{1.3D2}$-Gal4; UAS-cd8GFP, UAS-dcr2	14
	**n**	GMR-myr mRFP; loco$^{1.3D2}$-Gal4; UAS-cd8GFP, UAS-dcr2	14
	**o**	loco$^{1.3D2}$-Gal4/UAS-FB1.1^{260b}; hs-mFLP5/+ (55h APF)	11
		loco$^{1.3D2}$-Gal4/UAS-FB1.1^{260b}; hs-mFLP5/+ (60h APF)	14
		loco$^{1.3D2}$-Gal4/UAS-FB1.1^{260b}; hs-mFLP5/+ (70h APF)	10
		loco$^{1.3D2}$-Gal4/UAS-FB1.1^{260b}; hs-mFLP5/+ (80h APF)	8
**Fig. 3**	**c**	loco$^{1.3D2}$-Gal4/+; UAS-cd8GFP, UAS-dcr2/+	30
	**d**	loco$^{1.3D2}$-Gal4/UAS-lapsyn$^{IR-KK102333}$; UAS-cd8GFP, UAS-dcr2/+	14/19
	**e**	loco$^{1.3D2}$-Gal4/UAS-lapsyn$^{IR-15658R1}$; UAS-cd8GFP, UAS-dcr2/+	8/13
	**f**	loco$^{1.3D2}$-Gal4/UAS-FB1.1^{260b}; hs-mFLP5/+	60[b]
	**g**	loco$^{1.3D2}$-Gal4 UAS-FB1.1^{260b}/UAS-lapsyn$^{IR-KK102333}$;UAS-dcr2/hs-mFLP5	22
**Fig. 4**	**c**	Mi{MIC}lapsyn^{MI01316}/CyO	3
	**d**	Mi{MIC}lapsyn^{MI01316}/CyO	4
	**e**	Mi{MIC}lapsyn^{MI01316}/CyO	8
	**f**	Mi{MIC}lapsyn^{MI01316}/CyO	11
	**g**	Mi{MIC}lapsyn^{MI01316}/CyO	6
	**h**	Mi{MIC}lapsyn^{MI01316}/CyO	5
	**i**	loco$^{1.3D2}$-Gal4/lapsyn$^{2xHA-CR13.1}$; UAS-cd8GFP/+	6
	**j**	lapsyn$^{fTRG027706-135B}$/+	6
**Fig. 5**	**a**	ey-FLP/+ or Y; FRT42B tubP-Gal80/loco$^{1.3D2}$-Gal4, FRT42B; UAS-cd8GFP/+	28
	**b**	ey-FLP/+ or Y; FRT42B tubP-Gal80/loco$^{1.3D2}$-Gal4, FRT42B lapsyn^{ZG1}; UAS-cd8GFP/+	29
	**c**	ey-FLP/+ or Y; FRT42B tubP-Gal80/loco$^{1.3D2}$-Gal4, FRT42B lapsyn^{LL00906}; UAS-cd8GFP/+	18
	**d**	loco$^{1.3D2}$-Gal4/+; UAS-cd8GFP/UAS-lapsyn	4
	**e**	ey-FLP/+ or Y; FRT42B tubP-Gal80/loco$^{1.3D2}$-Gal4, FRT42B; UAS-cd8GFP/UAS-lapsyn	16
	**f**	ey-FLP/+ or Y; FRT42B tubP-Gal80/loco$^{1.3D2}$-Gal4, FRT42B lapsyn^{ZG1}; UAS-cd8GFP/UAS-lapsyn	15
	**i**	wrapper^{GMR54H02}-Gal4 UAS-myr mRFP/+	13
	**j**	UAS-lapsyn $^{IR-KK102333}$/+; wrapper^{GMR54H02}-Gal4 UAS-myr mRFP/UAS-dcr2	15
	**k**	wrapper^{GMR54H02}-Gal4 UAS-myr mRFP/UAS-lapsyn	14
**Fig. 6**	**a**	ey-FLP/+ or Y; FRT42B tubP-Gal80/loco$^{1.3D2}$-Gal4, FRT42B; UAS-cd8GFP/+	19
	**b**	ey-FLP/+ or Y; FRT42B tubP-Gal80/loco$^{1.3D2}$-Gal4, FRT42B; UAS-cd8GFP/+	13
	**c**	ey-FLP/+ or Y; FRT42B tubP-Gal80/loco$^{1.3D2}$-Gal4, FRT42B; UAS-cd8GFP/+	28
	**d**	ey-FLP/+ or Y; FRT42B tubP-Gal80/loco$^{1.3D2}$-Gal4, FRT42B lapsyn^{ZG1}; UAS-cd8GFP/+	12
	**e**	ey-FLP/+ or Y; FRT42B tubP-Gal80/loco$^{1.3D2}$-Gal4, FRT42B lapsyn^{ZG1}; UAS-cd8GFP/+	12

	f	ey-FLP/+ or Y; FRT42B tubP-Gal80/loco$^{1.3D2}$-Gal4, FRT42B lapsyn^{ZG1}; UAS-cd8GFP/+	29
	g	ey-FLP/+ or Y; FRT42B tubP-Gal80/loco$^{1.3D2}$-Gal4, FRT42B; UAS-cd8GFP/+	19
	h	ey-FLP/+ or Y; FRT42B tubP-Gal80/loco$^{1.3D2}$-Gal4, FRT42B; UAS-cd8GFP/+	6
	i	ey-FLP/+ or Y; FRT42B tubP-Gal80/loco$^{1.3D2}$-Gal4, FRT42B; UAS-cd8GFP/+	18
	j	ey-FLP/+ or Y; FRT42B tubP-Gal80/loco$^{1.3D2}$-Gal4, FRT42B lapsyn^{ZG1}; UAS-cd8GFP/+	26
	k	ey-FLP/+ or Y; FRT42B tubP-Gal80/loco$^{1.3D2}$-Gal4, FRT42B lapsyn^{ZG1}; UAS-cd8GFP/+	12
	l	ey-FLP/+ or Y; FRT42B tubP-Gal80/loco$^{1.3D2}$-Gal4, FRT42B lapsyn^{ZG1}; UAS-cd8GFP/+	21
	o	ey-FLP/+ or Y; FRT42B tubP-Gal80/loco$^{1.3D2}$-Gal4, FRT42B; UAS-cd8GFP/+	29
	p	ey-FLP/+ or Y; FRT42B tubP-Gal80/loco$^{1.3D2}$-Gal4, FRT42B lapsyn^{ZG1}; UAS-cd8GFP/+	11
**Fig. 7**	a	htl^{fTRG482} (42h APF)	7
		htl^{fTRG482} (55h APF)	4
		htl^{fTRG482} (72h APF)	4
	b	wild type^{OreR} (42h APF)	8
		wild type^{OreR} (55h APF)	13
		wild type^{OreR} (72h APF)	16
	c	Mi{MIC}ths^{MI01564}/CyO	6
	d	loco$^{1.3D2}$-Gal4/+; UAS-cd8GFP/+	15
	e	loco$^{1.3D2}$-Gal4/UAS-htl$^{IR\text{-}GD6692}$; UAS-cd8GFP/+	5
	f	loco$^{1.3D2}$-Gal4/UAS-htl$^{IR\text{-}GD27180}$; UAS-cd8GFP/+	33
	g	loco$^{1.3D2}$-Gal4/+; UAS-cd8GFP/UAS-htl$^{\lambda}$	6
	i	loco$^{1.3D2}$-Gal4/+; UAS-cd8GFP/+ (24h)	14
		loco$^{1.3D2}$-Gal4/+; UAS-cd8GFP/UAS-htl$^{\lambda}$ (24h)	14
	k	loco$^{1.3D2}$-Gal4/+; UAS-cd8GFP/+	10
		loco$^{1.3D2}$-Gal4/+; UAS-cd8GFP/UAS-htl$^{\lambda}$	11
**Fig. 8**	a	loco$^{1.3D2}$-Gal4/UAS-htl$^{IR\text{-}GD27180}$; UAS-cd8GFP/+	33[c]
	b	loco$^{1.3D2}$-Gal4/UAS-htl$^{IR\text{-}GD27180}$; UAS-cd8GFP/UAS-lapsyn	16
	d	ey-FLP/+ or Y; FRT42B tubP-Gal80/loco$^{1.3D2}$-Gal4, FRT42B; UAS-cd8GFP/+	14
	e	ey-FLP/+ or Y; FRT42B tubP-Gal80/loco$^{1.3D2}$-Gal4, FRT42B; UAS-cd8GFP/UAS-htl$^{\lambda}$	16
	f	ey-FLP/+ or Y; FRT42B tubP-Gal80/loco$^{1.3D2}$-Gal4, FRT42B lapsyn^{ZG1}; UAS-cd8GFP/UAS-htl$^{\lambda}$	14
**Fig. 9**	a	lapsyn$^{fTRG027706\text{-}135B}$	9
	b	loco$^{1.3D2}$-Gal4; UAS-cd8GFP	10
	c	loco$^{1.3D2}$-Gal4/UAS-lapsyn$^{IR\text{-}KK102333}$; UAS-cd8GFP/UAS-dcr2	6
	d	lapsyn$^{fTRG027706\text{-}135B}$	8
	e	loco$^{1.3D2}$-Gal4; UAS-cd8GFP	14
	f	loco$^{1.3D2}$-Gal4/UAS-lapsyn$^{IR\text{-}KK102333}$; UAS-cd8GFP/UAS-dcr2	11
	g	lapsyn$^{fTRG027706\text{-}135B}$	10
	h	loco$^{1.3D2}$-Gal4; UAS-cd8GFP	11
	i	loco$^{1.3D2}$-Gal4/UAS-lapsyn$^{IR\text{-}KK102333}$; UAS-cd8GFP/UAS-dcr2	10
**Suppl. Fig. 1**	a	repo-Gal4/UAS-cd8GFP	4
	b	NP6520-Gal4/UAS-cd8GFP	4
	c	alrm-Gal4/+; UAS-cd8GFP/+	4
	d	alrm-Gal4/UAS-cd8GFP	7
	e	loco$^{1.3D2}$-Gal4/+; UAS-cd8GFP/+	4
	f	loco$^{1.3}$-lexA/lexAop-cd8GFP	5
	g	R56F03-Gal4/UAS-FB1.1B^{260b}; loco$^{1.3}$-LexA^{attP2} LexAop-myr mCherry/+	16
	h	R56F03-Gal4/UAS-FB1.1B^{260b}; loco$^{1.3}$-LexA^{attP2} LexAop-myr mCherry/+	16
**Suppl. Fig. 2**	a	loco$^{1.3D2}$-Gal4; UAS-cd8GFP	13
	b	loco$^{1.3D2}$-Gal4; UAS-cd8GFP	12
	c	dEAAT1-Gal4/+; UAS-cd8GFP/+	6
	d	loco$^{1.3D2}$-Gal4; UAS-cd8GFP	12

**Suppl. Fig. 3**	**a**	*loco*$^{1.3D2}$*-Gal4/UAS-ChRFP-Tub; UAS-cd8GFP/+*	8
	**b**	*loco*$^{1.3D2}$*-Gal4, UAS-cd8mCherry*^{260b}*/+; UAS-Eb1-GFP/+*	32
	**c**	*loco*$^{1.3D2}$*-Gal4, UAS-cd8mCherry*^{260b}*/+; UAS-nod-GFP/+*	9
	**d**	*loco*$^{1.3D2}$*-Gal4, UAS-cd8mCherry*^{260b}*/+; UAS-LifeAct-GFP/+*	20
	**e**	*loco*$^{1.3D2}$*-Gal4, UAS-cd8mCherry*^{260b}*/UAS-lapsyn*$^{IR\text{-}KK102333}$*; UAS-Eb1-GFP/UAS-dcr2*	12
	**f**	*loco*$^{1.3D2}$*-Gal4, UAS-cd8mCherry*^{260b}*/UAS-lapsyn*$^{IR\text{-}KK102333}$*; UAS-LifeAct-GFP/UAS-dcr2*	8
**Suppl. Fig. 4**	**c**	*loco*$^{1.3D2}$*-Gal4/Mi{MIC}lapsyn*$^{lapsyn\text{-}2xHA\text{-}24A}$*; UAS-cd8GFP/+*	19
	**d**	*loco*$^{1.3D2}$*-Gal4/+; UAS-lapsyn-GFP/+*	4
**Suppl. Fig. 5**	**a**	*ey-FLP/+* or *Y; FRT42B tubP-Gal80/loco*$^{1.3D2}$*-Gal4, FRT42B; UAS-cd8GFP/loco*$^{1.3}$*-lexA, lexAop-myr mCherry*	5
	**b**	*ey-FLP/+* or *Y; FRT42B tubP-Gal80/loco*$^{1.3D2}$*-Gal4, FRT42B; UAS-cd8GFP/loco*$^{1.3}$*-lexA, lexAop-myr mCherry*	11
	**c**	*ey-FLP/+* or *Y; FRT42B tubP-Gal80/loco*$^{1.3D2}$*-Gal4, FRT42B lapsyn*^{ZG1}*; UAS-cd8GFP/loco*$^{1.3}$*-lexA, lexAop-myr mCherry*	7
	**d**	*ey-FLP/+* or *Y; FRT42B tubP-Gal80/loco*$^{1.3D2}$*-Gal4, FRT42B lapsyn*^{ZG1}*; UAS-cd8GFP/loco*$^{1.3}$*-lexA, lexAop-myr mCherry*	13
**Suppl. Fig. 6**	**a**	*ey-FLP/+* or *Y; FRT42B tubP-Gal80/loco*$^{1.3D2}$*-Gal4, FRT42B; UAS-cd8GFP/UAS-p35*	13
	**b**	*ey-FLP/+* or *Y; FRT42B tubP-Gal80/ loco*$^{1.3D2}$*-Gal4, FRT42B lapsyn*^{ZG1}*; UAS-cd8GFP/UAS-p35*	13
	**c**	*GMR-myr mRFP/+* or *Y; loco*$^{1.3D2}$*-Gal4/+; UAS-cd8GFP/+*	4
	**d**	*GMR-myr mRFP/+* or *Y; loco*$^{1.3D2}$*-Gal4/UAS-htl*$^{IR\text{-}GD27180}$*; UAS-cd8GFP/+*	4
	**e**	*loco*$^{1.3D2}$*-Gal4/+; UAS-cd8GFP/+*	3
	**f**	*loco*$^{1.3D2}$*-Gal4/UAS-htl*$^{IR\text{-}GD27180}$*; UAS-cd8GFP/+*	3
**Suppl. Fig. 7**	**a**	*loco*$^{1.3D2}$*-Gal4/+; UAS-cd8GFP/UAS-htl*$^{\lambda}$	3
	**b**	*loco*$^{1.3D2}$*-Gal4/+; UAS-cd8GFP/UAS-htl*$^{\lambda}$*, UAS-lapsyn*	4
**Suppl. Fig. 9**	**c**	*lapsyn*$^{fTRG027706\text{-}135B}$	9
	**d**	*lapsyn*$^{fTRG027706\text{-}135B}$	10
	**e**	*loco*$^{1.3D2}$*-Gal4; UAS-cd8GFP*	19
	**f**	*loco*$^{1.3D2}$*-Gal4/UAS-lapsyn*$^{IR\text{-}KK102333}$*; UAS-cd8GFP/UAS-dcr2*	14

[a] number of cell types observed: long dmng, n=57 (**1d**,**g**,**h**); short dmng, n=35 (**1i**,**j**); lmng, n= 12 (**1f**); pmng, n=7 (**1e**).

[b] same n number as in Figure **1e**–**k**.

[c] from same data set as in Figure **7f**.

Supplementary Table 2 | Additional statistical values of quantifications in main and supplementary figures

Figure	Panel	t=, df= (pairwise comparisons from left to right)
**Fig. 4**	**b**	Welch corrected, not assuming equal SD:<br>Comparison 1: t=0.167 df=2.78<br>Comparison 2a: t=2.627 df=2.317<br>Comparison 2b: t=0.474 df=2.119<br>Comparison 3a: t=7.152 df=2.949<br>Comparison 3b: t=0.088 df=2.327<br>Comparison 4a: t=5.994 df=3.914<br>Comparison 4b: t=0.0107 df=3.327<br>Comparison 5a: t=14.509 df=2.000<br>Comparison 5b: t=0.183 df=2.854
**Fig. 5**	**h**	Welch corrected, not assuming equal SD:<br>Comparison 1: t=9.635 df=42.56<br>Comparison 2: t=4.917 df=63.93<br>Comparison 3: t=1.732 df=62.49<br>Comparison 4: t=1.258 df=61.97
**Fig. 6**	**m**	Welch-corrected, not assuming equal SD:<br>Comparison 1: t=2.571 df=54.44<br>Comparison 2: t=1.908 df=25.16<br>Comparison 3: t=4.136 df=47.30<br>Comparison 4: t=4.396 df=52.00<br>Comparison 5: t=5.946 df=43.00<br>Comparison 6: t=9.743 df=42.72
**Fig. 6**	**n**	U:<br>374.5<br>47<br>148<br>195
**Fig. 6**	**q**<br>left<br>right	U:<br>93.5<br>Comparison 1: 227<br>Comparison 2: 54<br>Comparison 3: 325.5
**Fig. 7**	**h**	Welch corrected, not assuming equal SD:<br>Comparison 1: t=10.78 df=35.42<br>Comparison 2: t=4.960 df=6.938<br>Comparison 3: t=2.620 df=6.416
**Fig. 7**	**j**	Welch corrected, not assuming equal SD:<br>Comparison 1: t=6.073 df=16.555<br>Comparison 2: t=2.881 df=5.248
**Fig. 7**	**l**	Welch corrected, not assuming equal SD:<br>t=4.799 df=10.170
**Fig. 8**	**c**	Welch corrected, not assuming equal SD:<br>Comparison 1: t=10.78 df=35.42<br>Comparison 2: t=8.538 df=29.50<br>Comparison 3: t=0.656 df=33.03
**Fig. 8**	**g**<br>top	Welch corrected, not assuming equal SD:<br>Comparison 1: t=2.169 df=25.580<br>Comparison 2: t=0.556 df=12.796<br>Comparison 3: t=2.406 df=24.229<br>Comparison 4: t=2.686 df=19.578<br>Comparison 5: t=5.347 df=47.683

		Comparison 6: t=5.068 df=38.518
	bottom	Welch corrected, not assuming equal SD:<br>Comparison 1: t=9.416 df=16.739<br>Comparison 2: t=6.5 df=31.135<br>Comparison 3: t=13.878 df=30.925<br>Comparison 4: t=1.595 df=9.574<br>Comparison 5: t=48.523 df=36<br>Comparison 6: t=11.995 df=8.972
**Suppl. Fig. 7**	**c**	Welch corrected, not assuming equal SD:<br>t=1.646 df=27.866
**Suppl. Fig. 7**	**d**	Welch corrected, not assuming equal SD:<br>t=4.803 df=23.32

Supplementary Table 3 | Additional information for genetic reagents used

Transgene	Reference
elav-Gal4^{C155}	Robinow & White, 1991 (ref. 1)
repo-Gal4	Sepp *et al.*, 2001 (ref. 2)
UAS-FB1.1B^{260b}	Shimosako *et al.*, 2014 (ref. 3)
lexAop-myr mCherry	Diegelmann *et al.* 2008 (ref. 4)
UAS-ChRFP-Tub	Rusan & Peifer, 2007 (ref. 5)
UAS-Eb1-GFP	Rolls *et al.*, 2007 (ref. 6)
UAS-nod-GFP	Andersen *et al.*, 2005 (ref. 7)
UAS-LifeAct-GFP	Zanet *et al.*, 2012 (ref. 8)
ey-FLP	Newsome *et al.*, 2000 (ref. 9)
UAS-p35	Hay *et al.*, 1994 (ref. 10)

Supplementary References

1. Robinow, S. & White, K. Characterization and spatial distribution of the ELAV protein during Drosophila melanogaster development. *J. Neurobiol.* **22**, 443-461 (1991).
2. Sepp, K.J., Schulte, J. & Auld, V.J. Peripheral glia direct axon guidance across the CNS/PNS transition zone. *Dev. Biol.* **238**, 47-63 (2001).
3. Shimosako, N., Hadjieconomou, D. & Salecker, I. Flybow to dissect circuit assembly in the Drosophila brain. *Methods Mol. Biol.* **1082**, 57-69 (2014).
4. Diegelmann, S., Bate, M. & Landgraf, M. Gateway cloning vectors for the LexA-based binary expression system in Drosophila. *Fly (Austin)* **2**, 236-239 (2008).
5. Rusan, N.M. & Peifer, M. A role for a novel centrosome cycle in asymmetric cell division. *J. Cell Biol.* **177**, 13-20 (2007).
6. Rolls, M.M.*, et al.* Polarity and intracellular compartmentalization of Drosophila neurons. *Neural Dev.* **2**, 7 (2007).
7. Andersen, R., Li, Y., Resseguie, M. & Brenman, J.E. Calcium/calmodulin-dependent protein kinase II alters structural plasticity and cytoskeletal dynamics in Drosophila. *J. Neurosci.* **25**, 8878-8888 (2005).
8. Zanet, J.*, et al.* Fascin promotes filopodia formation independent of its role in actin bundling. *J. Cell Biol.* **197**, 477-486 (2012).
9. Newsome, T.P., Asling, B. & Dickson, B.J. Analysis of Drosophila photoreceptor axon guidance in eye-specific mosaics. *Development* **127**, 851-860 (2000).
10. Hay, B.A., Wolff, T. & Rubin, G.M. Expression of baculovirus P35 prevents cell death in Drosophila. *Development* **120**, 2121-2129 (1994).
